# Virtual Reality Simulation for Pediatric Airway Intubation Readiness Education

**DOI:** 10.7759/cureus.12059

**Published:** 2020-12-13

**Authors:** Nisha Agasthya, Scott Penfil, Nicholas Slamon

**Affiliations:** 1 Pediatric Critical Care, Kansas University School of Medicine, Wichita, USA; 2 Pediatric Critical Care, Herman & Walter Samuelson Children’s Hospital at Sinai, Baltimore, USA; 3 Pediatric Critical Care, Nemours/Alfred I. duPont Hospital for Children, Wilmington, USA

**Keywords:** virtual reality, simulation, pediatric, airway intubation, medical education

## Abstract

Background

The objective of this study was to provide education to inexperienced trainees regarding preparation for airway intubation using virtual reality (VR) tutorial and comparison of performance with that of experienced trainees without VR training. We hypothesized that after the VR tutorial, junior fellows and residents will have comparable recall of the proper steps as experienced trainees.

Methods

This project was initiated in the pediatric intensive care unit from July 1, 2019, to July 30, 2019. Volunteer residents and pediatric critical care medicine fellows participated. The VR group completed a 19-minute immersive tutorial and then demonstrated learned skills with a traditional manikin. Non-VR group fellows listed steps to prepare for airway intubation from memory with scoring on a 24-point timed checklist.

Results

Seventeen subjects participated; two residents were excluded. The VR group had seven trainees (47%) and scored similarly to the other group based on checklist items (50.5% vs 50.8%, P=1).

Conclusion

VR technologies can be used for education in preparation for pediatric airway intubation. There was no difference in the performance accuracy between the two groups. Larger studies are essential to study benefits of VR in preparation and performance of airway intubation.

## Introduction

Mastery of pediatric airway management is an important skill for trainees from various specialties. Preparation for pediatric intubation involves familiarity with and education on the nuances of the pediatric airway including anatomy and proper patient positioning; correct equipment choices; and use and set-up as well as knowledge of various scenarios that may affect the choice of sedative, analgesic, and neuromuscular blockade. The knowledge of these steps with repetitive training and meticulous pre-procedure preparation can help mitigate difficulties encountered in the process of airway intubation and prevent adverse outcomes.

Traditional medical education has a didactic approach, which is often difficult to implement when learning procedural skills and has been shown to be ineffective for adult learners in medicine. In 2002, a working group of experts concluded that simulation-based education is an optimal modality for learning [1]. Simulation lends itself to a see, do, and teach approach that allows for a combination of learning styles that leads to better retention. Learners are able to see an instructor demonstrate the procedure, and then experience the act using their tactile and visual senses. To cement their learning, they then can teach that procedural act to another. A small number of studies have demonstrated that simulation-based medical education with deliberate practice can be superior to traditional clinical education [2]. 

However, traditional manikin-based simulation can lack realism, require extensive set-up and dedicated staff, and is limited in the number of learners who can access the simulation center at one time [3]. Computer-based technologies have evolved at a rapid pace with virtual reality (VR) used in various aspects of medical education including surgical training, decision-making skills in trauma, and cardiopulmonary resuscitation training [4-6]. VR provides a realistic, immersive, on-demand experience. It can be customized to each user, and includes wearing a headset for immersive interaction with a software environment. In addition, recent technological developments in video conferencing allow for the participation of multiple users, even those without their own VR headsets, to experience simulation remotely while still being able to practice team training and closed-loop communication from anywhere. It has been used in training for performing orotracheal and fiber-optic intubation [7-9]. What is not well understood is if a single instance of VR training as described can rapidly advance the knowledge of a novice to a similar level of training of more experienced physicians when assessing the steps needed for intubation preparation.

In this study, we incorporated VR technology for the education of novice pediatric residents and critical care medicine fellows on the sequential steps required to prepare for a pediatric airway intubation. We hypothesized that inexperienced pediatric trainees after undergoing a brief immersive VR tutorial would have comparable performance with experienced senior fellows and emergency medicine residents when asked to demonstrate learned skills on a traditional manikin.

This article was previously presented as a meeting abstract at the 2020 SCCM Critical Care Congress, Orlando, FL, on February 16, 2020.

## Materials and methods

Design, setting, and participants

This was a prospective randomized comparison study initiated from July 1 to 31, 2019, in the pediatric intensive care unit (PICU) of a quaternary children’s hospital with Accreditation Council for Graduate Medical Education (ACGME)-accredited pediatric residency and subspecialty (fellowship) training programs. Pediatric residents (postgraduate year [PGY] 2), visiting emergency medicine (PGY 2) residents completing clinical rotation in the PICU during the study period, and pediatric critical care fellows (PGYs 4, 5, 6) participated in this project.

**Figure 1:**
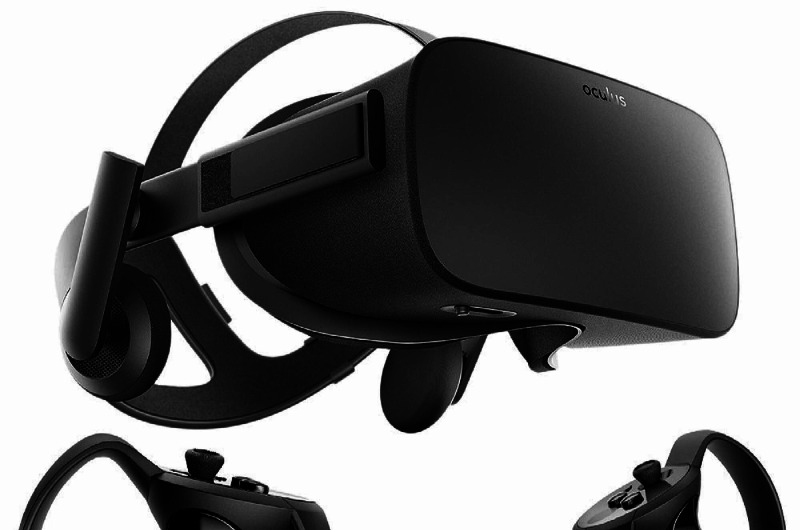
Head-mounted set with hand controls

The VR technology used included Oculus Rift S headset with hand controls (Facebook LLC, San Jose, CA) (Figure 1) and an Alienware gaming laptop (Dell Technologies, Round Rock, TX). The VR simulation software tutorial for pediatric airway intubation was developed using the Acadicus simulation platform (Arch Virtual, Madison, WI).

Study groups

**Figure 2:**
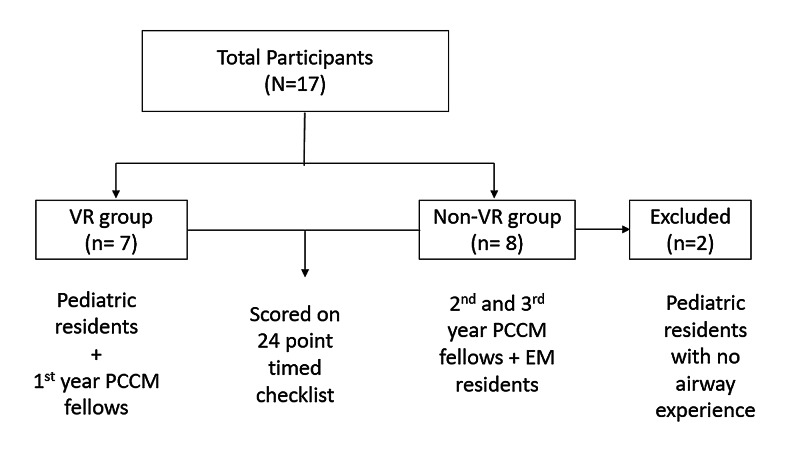
Flowchart of study groups PCCM: pediatric critical care medicine; EM: emergency medicine

Two study groups were randomized (n=17) (Figure 2). Pediatric residents and first-year fellows (n=7) were included in the VR group. Upper-year fellows (PGYs 5 and 6) and emergency medicine residents (n=8) were included in the non-VR group. Two pediatric residents in the non-VR group were excluded for study analysis given their inexperience with airway management. They were unavailable the day the VR training was offered but still wished to participate in the study scoring.

**Figure 3:**
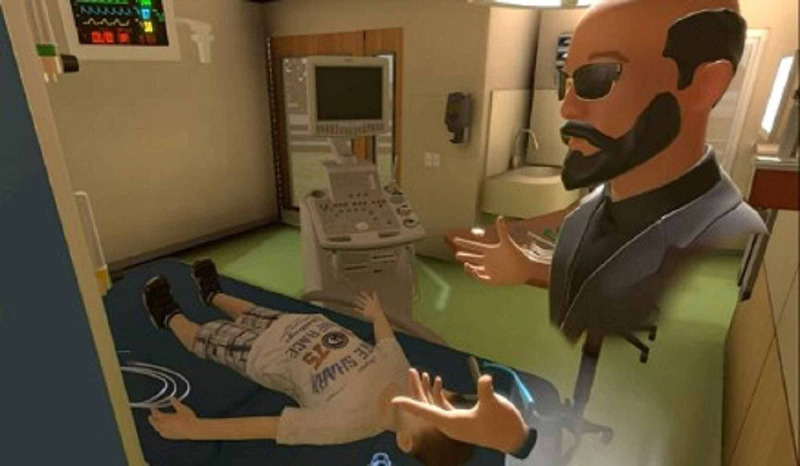
Image from VR application showing the avatar-based tutorial layout VR: virtual reality

The VR group completed a 19-minute immersive tutorial that outlined the steps involved in preparation for pediatric airway intubation. An interactive avatar in the VR tutorial (Figure 3) discussed the steps in detail in a room created to replicate the PICU’s patient room.

**Figure 4:**
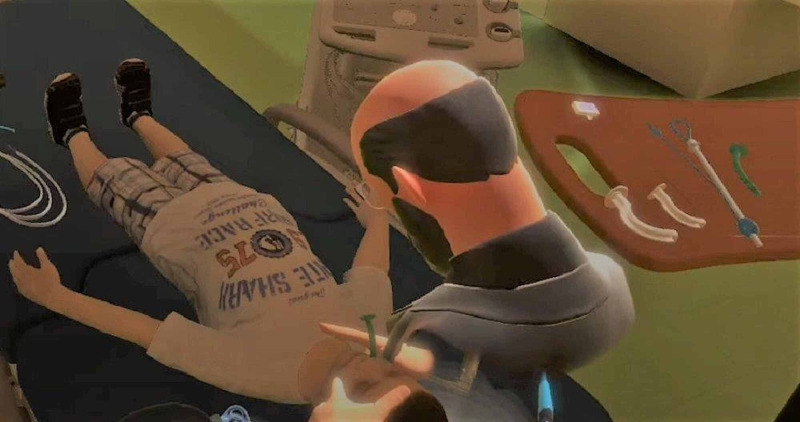
Avatar showing the placement of nasopharyngeal airway

In addition to watching the tutorial in the VR room from multiple angles as they could move around freely in the simulated environment, they were also able to visualize, pick up, and utilize equipment in the VR environment such as squeezing the bag and mask to deliver breaths, placement of nasopharyngeal airway, laryngoscopy, and endotracheal tube insertion (Figure 4). After completing their time in the virtual environment, the VR group was asked to demonstrate the learned steps on a traditional manikin and verbally announce each steps as they performed them. The non-VR group listed the steps in the airway preparation process from memory without additionally being asked to demonstrate the steps on the manikin. Both groups were scored on a 24-point timed checklist (Table 1) by the same observer who is a critical care attending physician. The checklist was prepared based on the most the common equipment and preparation required pre-intubation by the study authors who have all completed pediatric critical care fellowship training.

**Table 1:** The 24-point testing checklist

Checklist items	
Bag mask size 1	Bag mask size 2
Tracheal tube size 1	Tracheal tube size 2
Check tracheal tube cuff	Stylette
Laryngoscopes	Check laryngoscope light
Oral airway	Nasopharyngeal airway
End-tidal CO_2 _detector	Mechanical ventilator
Nasogastric tube	Rigid suction catheter
Flexible suction catheter	Place patient on monitor
Cycle blood pressure cuff	Remove head board
Lower side rails	Peripheral IV confirmation
Wash hands	Wear gloves
Patient positioning	Gather personnel

Statistical analysis

Statistical analysis was performed using SPSS Statistics software (IBM Corp., Armonk, NY), and Mann-Whitney U test was used for data analysis.

## Results

The VR group included seven participants (47%). They scored similarly to the non-VR group on the checklist items after completion of the tutorial (50.5% vs 50.8%, P=1). Some of the steps missed most frequently by the VR group included failing to request an end-tidal carbon dioxide detector (42%), choosing a variety of nasopharyngeal airways (57%), failure to request a nasogastric tube (100%), failure to request set-up of repetitive cyclic blood pressure measurements during subsequent intubation (42%), and failure to request nursing confirmation of functioning peripheral intravenous access (57%). The median time to complete the set-up steps was higher in the VR group (6 vs 3.5 minutes, P=0.005). When allowing for requests for advanced airway equipment and medications not covered in the VR tutorial, such as fiberoptic scope or laryngeal mask airway (73.6% vs 55.3%, P=0.0009), the non-VR group scored higher.

## Discussion

In this study, an immersive, VR tutorial-based learning module taught residents and first-year pediatric critical care fellows the steps involved in the preparation for airway intubation. While this study was not focused on the performance of the tracheal intubation itself, preparedness by having all the necessary equipment available can help with the success of the procedure itself. The act of setting up is an important skill for trainees who perform this procedure in the intensive care unit and emergency department. The number of tracheal intubation attempts is associated with adverse events [10]. Adequate education on training and preparation may help mitigate adverse events like oxygen desaturation, hypotension, and cardiorespiratory arrest. This fellowship training program provides training via lecture-based and practice via manikin-based methods to residents and fellows.

VR learners showed no statistical difference in accuracy when compared to experienced fellows after participation in the immersive tutorial. This suggests VR technology maybe utilized for this purpose. The ability to recreate this training in a VR format offers several advantages over traditional boot camp style teaching or pre-recorded watchable video tutorials. VR training gives learners access to a three-dimensional, immersive setting that contains the equipment and replicates the environment they are being asked to work in without having to reserve space in the simulation lab or intensive care unit for practice sessions. It allows an instructor to pre-record a demonstration in which the learner can not only watch the steps, but play an active role in the tutorial as well. The learner can use equipment and repeat the steps of the recorded instructor who is free to do other tasks. Learners can also complete their tutorial at their own pace and in their own time and repeat the exercise as many times as they choose until reaching a level of comfort with the skills.

The time to execute the steps for set-up in the physical simulation lab by the VR group (after their VR tutorial was complete) was compared to the verbal report of the steps by the advanced fellows. Although there was a statistically significant result, it offers several insights that probably invalidate its use in this study. First, the VR group not only called out their steps but they also went on to demonstrate its use on the manikin in the simulation lab. The advanced fellows simply recited without subsequent demonstration. In addition, as previously discussed the VR tutorial was focused on gathering and testing of the equipment that could be needed for intubation, and not the procedure itself. One could argue that gathering the equipment quickly in an emergent clinical scenario is important and this time difference could be clinically relevant. However, in reality, the intensive care unit is a team environment with support staff available to assist in equipment procurement at most times. The key to this VR training exercise was to familiarize the learner with the sequential steps in equipment set-up.

When allowing the advanced fellows to “freelance” during their intubation set-up exercise, they requested equipment that was not on the checklist. Although the score was significantly higher than that for the VR participants, the tutorial was intended to cover basic tenets of airway preparation. It is a consideration to make a second advanced airway preparation tutorial that creates assets not yet present in the VR environment. This likely comes from their collective experiences over the first two years of fellowship with the challenging clinical scenarios.

Over the past five years, VR technologies have been rapidly advancing. Improvements in graphics, headset resolution, and ability of hardware to refresh images quickly leading to a seamless appearance of the environment have made the experience of VR move from an awkward cartoonish endeavor to one where it has become difficult to separate it from reality. Its extension into medical simulation has been led by companies interested in training surgeons, in particular, to practice operations in a way that mimics reality but puts no patient at harm.

The success of surgical VR has paved the way for the proliferation of immersive medical simulations that feature rare diseases like high-altitude cerebral edema (SimX, Mountain View, CA), common diseases such as sepsis in an oncology patient (Oxford Medical Simulation, Boston, MA), and interactive tutorials with customizable environments and equipment (Arch Virtual, Madison, WI). A significant reduction in cost and space requirements and enhanced realism when compared with traditional simulation centers have made these systems increasingly popular. Their rising popularity and expansion have been seen over the past several years at the International Meeting on Simulation in Healthcare (IMSH) with more companies and new features being showcased each year.

Zackoff et al. demonstrated in their study that a VR curriculum focused on pediatric respiratory failure can help trainees recognize and interpret changes in clinical status compared with traditional high-fidelity, manikin-based simulation based on self-assessment of competence [11].

There are several advantages of VR application in medical education. It is portable, is simple to set up, and requires very little space. An area as small as 12 square feet can support a simulation when using a VR headset. This allows for financial flexibility that makes simulation more accessible to health systems that do not have dedicated laboratories. It also offers options to systems whose simulation lab space and time is overbooked or expensive to maintain. The learner is also able to navigate the scenario without the support of additional staff, thus limiting additional restrictions in time and scheduling.

The scenarios are repeatable and allow learners to practice skills until comfortable. An improvement in performance occurs at times that are convenient for the learner [12]. Multiple users can also interact within the same platform from great distances while still fostering team dynamics. Avatar-mediated learning has been shown to improve communication skills and has been shown to be effective in pediatrics [13,14]. The immersive nature of this technology can make user experience more satisfactory than traditional learning. A survey of medical students showed that the process of using VR was enjoyable and improved confidence with the training received [15]. Recent improvements in video conferencing also allow for an increase in the number of users who can benefit from a VR session even when they do not own a VR headset and computer. With a singular headset and laptop, a VR scenario can be streamed live to multiple participants using teleconferencing platforms such as Zoom (Zoom Video Communications, Inc., San Jose, CA), Webex (Cisco Webex, Milpitas, CA), or Microsoft Teams (Microsoft Corp., Redmond, Washington). Remote participants can see and hear the action in the scenario and direct the in-scenario avatar remotely. They can collaborate and practice team mechanics, closed-loop communication, and debrief from multiple points on the globe simultaneously. The recent closure of most simulation centers because of the global coronavirus disease 2019 (COVID-19) pandemic makes VR an attractive option to conduct team training in medical simulation. Given the global physician shortage in the times of pandemic, VR use is helping training medical students and out-of-practice physicians to gain skills and competence [16]. The validity of its use in this situation is yet to be examined.

The cost of simulation-based medical education is underreported in the literature [17]. One study reported an estimated $100,000 to set up a basic simulation lab, while an advanced center can have costs in the millions with the annual maintenance costing at least $15,000 [18,19]. However, the practical aspects of acquiring and maintaining high-fidelity manikins can be overcome using VR. Technology-based simulation costs include headset, computer laptop, and software subscription, which are comparatively less expensive.

This technology comes with disadvantages as well. Many aspects of clinical medicine have not been replicated and, at this time, require in-person, bedside teaching. The ability of experiencing tactile perception like that of passing a guidewire through a blood vessel or displacement of the epiglottis during airway intubation can be difficult to mimic and has yet to be simulated using haptic feedback. In addition, the development of haptic features is still in its infancy and, thus, comes with a significant financial burden at this time. However, there have been recent randomized controlled trials comparing VR with haptic technology and those without in surgical procedures. These trials have shown promise. VR simulation with haptic feedback can enhance user experience with force-feedback mechanisms and improve realism in skills like cutting, suturing, and dissection [20]. While this software tutorial did not include haptic technology, addition of this feature may be useful when physical distancing is encouraged. Familiarity and comfort with newer technology is another potential disadvantage. Finally, there may be an indirect increase in the cognitive load in VR simulation that may negatively affect learning and must be studied further [21].

Limitations

This study as it has been conducted has several limitations. The sample size of participants is small. This is the experience of a single academic center where simulation-based education is with the use of manikins with no previous experience in VR. The non-VR participants were tested on accuracy by recall method and compared to the VR group who were asked to recall and demonstrate on a manikin. The tutorial also did not include additional airway adjuncts that were mentioned by the experienced fellows, which will need to be added in future iterations of the software.

## Conclusions

In this study, there were no differences in the accuracy of performance while learning the steps to prepare for airway intubation between inexperienced learners who used VR technology and experienced senior fellows. VR-based remote tutorials may offer advantages over traditional simulation training and may continue to be necessary due to physical distancing requirements during the current and future global pandemics. A larger, randomized, multicenter study in VR training is required to show consistent benefit of VR simulation as a teaching tool in medical education.
